# Effectiveness of a novel orally administered combination drug product containing milbemycin oxime and lotilaner (Credelio^®^ Plus) for the treatment of larval and immature adult stages of *Ancylostoma caninum* in experimentally infected dogs

**DOI:** 10.1186/s13071-021-04761-y

**Published:** 2021-05-17

**Authors:** Daniel E. Snyder, Scott Wiseman, Elizabeth Crawley, Kim Wallace, Dwight D. Bowman, Craig R. Reinemeyer

**Affiliations:** 1 Elanco Animal Health Research and Development, 2500 Innovation Way, Greenfield, IN 46140 USA; 2 Elanco Animal Health, Priestley Road Basingstoke, Hants, RG24 9NL UK; 3 Daniel E. Snyder DVM PhD. Consulting, LLC, Indianapolis, IN 46229 USA; 4 College of Veterinary Medicine, Cornell University, Ithaca, NY USA; 5 East Tennessee Clinical Research Inc., Rockwood, TN USA

**Keywords:** *Ancylostoma caninum*, Chemoprophylaxis, Credelio Plus, Dog, Effectiveness, Hookworm, Immature, Larval, Lotilaner, Milbemycin oxime, Oral, Nematodes

## Abstract

**Background:**

The hookworm, *Ancylostoma caninum*, is a common and important zoonotic intestinal nematode parasite that infects dogs globally. Both the immature and adult stages of *A. caninum* ingest large volumes of blood during the feeding process and can cause severe anemia and death in young dogs, even before patent infections can be diagnosed using routine faecal examination methods. Thus, effective treatment of any pre-patent stages of immature hookworms can reduce or eliminate the risk of clinical disease in infected dogs and additionally reduce environmental contamination of eggs and infective larvae. Two randomized, blinded, GCP-compliant, pivotal laboratory dose confirmation studies were conducted to evaluate the effectiveness and safety of a new novel combination of lotilaner and milbemycin oxime tablets (Credelio Plus^®^) administered orally to dogs experimentally infected with immature (L4 and immature adult [L5]) stages of *A. caninum.*

**Methods:**

Treatments using the intended global commercial tablet formulation of Credelio Plus were administered in a time frame relative to inoculation with infective larvae so that effectiveness could be assessed against each specific immature stage of *A. caninum*. In each study, dogs were randomized to one of six (study 1) or four (study 2) treatment groups. Each treatment group contained 8 (study 1) or 10 (study 2) dogs that had been experimentally inoculated with infective *A. caninum* larvae on day 0 and were dosed once on day 7 or day 11. Enrolled subjects were administered placebo tablets, Credelio Plus tablets, or lotilaner mono tablets to provide minimum dosages of 0.75 mg/kg of milbemycin oxime and 20 mg/kg of lotilaner. All dogs were necropsied 5 days after their respective treatment. All nematodes recovered from the gastrointestinal tract at necropsy were counted by species and stage.

**Results:**

For both dose confirmation studies and based on geometric mean worm counts, efficacy of Credelio Plus was ≥ 97.3% against L4 larval stage of *A. caninum* and ≥ 98.7% against immature adult (L5) *A. caninum*.

**Conclusions:**

These studies demonstrated that the orally administered Credelio Plus combination tablet was highly efficacious in treating immature (L4 and immature adult [L5]) stages of *A. caninum* in experimentally infected dogs.
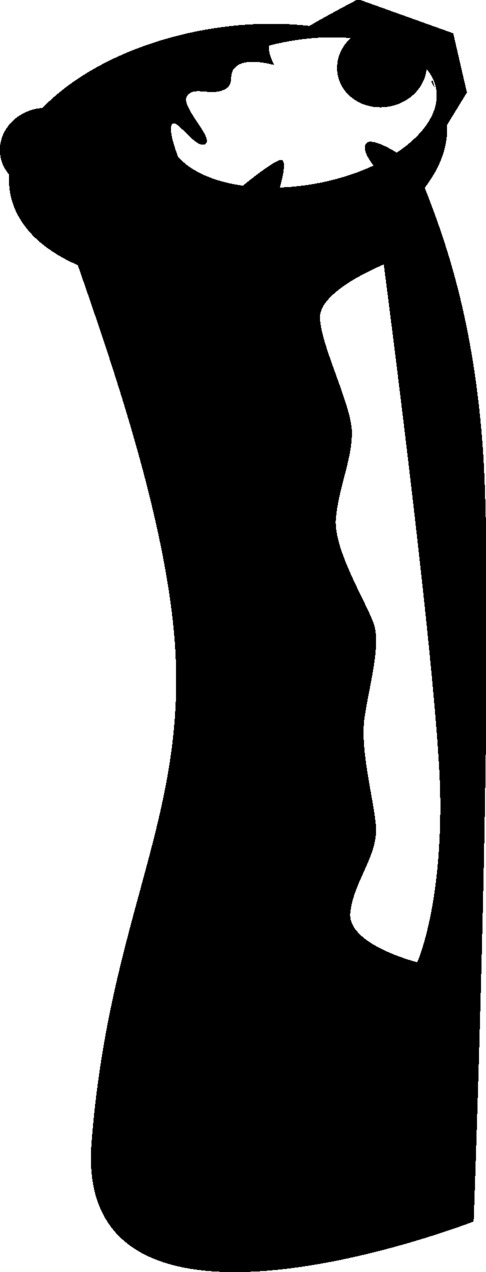

## Background

Intestinal nematode infections are commonly diagnosed in dogs from all parts of the world, including Europe [1–4]. Of these intestinal nematodes, *Ancylostoma caninum* and *Toxocara canis* are considered two of the most important and commonly reported helminth parasites of dogs [5, 6]. Dogs infected with *A. caninum* hookworm can play an important role in the transmission of this potentially zoonotic nematode by excreting eggs directly into the environment where humans and other dogs can be subsequently exposed to infective larvae. The veterinary and public health aspects of hookworm infections in dogs are well established [5]. In Europe, *A. caninum* and *Uncinaria stenocephala* are two hookworm species routinely found in dogs; however, *A. caninum* is reported to be found predominantly in dogs located in southern Europe [7].

Endectocidal combination products or anthelmintics with a broad spectrum of activity can provide the pet owner and veterinarian with the ability to treat dogs that are concurrently infested or infected with multiple parasite types. As reported herein, the combination of milbemycin oxime (MO) and lotilaner is one such example. The effectiveness of MO in combination with other drug substances administered to dogs that were naturally infected with different species of adult intestinal nematodes has been previously demonstrated in laboratory dose confirmation studies [8]. Additionally, it has been shown that a minimum dosage of 0.75 mg/kg of MO will prevent the establishment of the adult stage of the French heartworm, *Angiostrongylus vasorum,* and larval and immature adult stages of *T. canis* and *A. caninum* [9, 10]. The second drug substance in this fixed combination, lotilaner, has demonstrated excellent effectiveness against several ectoparasites (i.e., fleas, ticks, mites) on dogs [11–24].

The objective of both dose confirmation studies as summarized herein was to confirm the dose and non-interference of lotilaner (alone or in combination) with milbemycin oxime and to evaluate the effectiveness when administered at approximately the lower end (0.75 to 1.11 mg/kg MO) of the proposed MO tablet unit dose range (0.75 to 1.53 mg/kg MO), in dogs experimentally infected with immature (L4 and immature adult [L5]) stages of *Ancylostoma caninum.*

## Methods

Use of the lotilaner and MO combination investigational product (IP) in this study was intended to evaluate a proposed commercial formulation and dose regimen for use in dogs under end-user conditions for the intended European and global intestinal nematode label indications. As such, both dose confirmation studies were negative-controlled, masked, randomized laboratory studies to evaluate the effectiveness and safety of a combination of lotilaner and MO chewable tablets (IP) administered orally to dogs experimentally inoculated with *A. caninum*. These studies were conducted in accordance with the principles of GCP as laid down in the VICH guidelines GL9, Good Clinical Practice (June 2000); The Efficacy Requirements for Anthelmintics: Overall Guidelines (VICH GL7); The World Association for the Advancement of Veterinary Parasitology (WAAVP) guidelines for evaluating the efficacy of anthelmintics for dog and cats; VICH GLl9 Effectiveness of Anthelmintic and Overall Guidelines: Specific Recommendation for Canines (June 2001) [25–28]. Study site personnel involved in making assessments of intestinal nematode effectiveness and safety were masked to treatment assignments.

### Animals

In both studies purpose-bred laboratory research beagles were sourced from a USDA licensed vendor. All dogs were confirmed upon arrival to be free of any intestinal nematodes. These dogs were a mix of male/female, 2–3 months of age when inoculated with hookworm third-stage (L3) infective larvae, and had a body weight range of 3.65–7.0 kg. All dogs were acclimated for a minimum of 14 days prior to inoculation with infective L3 stages of *A. caninum* on day 0. All dogs were singly housed throughout the study in concrete floored pens that were cleaned daily and that restricted contact between dogs in adjacent pens, they were fed a balanced commercial dry dog food daily and provided with water ad libitum. Dogs were individually identified by ear tattoo. Physical examinations were performed during the acclimation period to ensure dogs were healthy and were eligible to be enrolled into each study. After day 0, all enrolled dogs were examined at least once daily for any abnormal clinical signs or adverse events. The primary inclusion criterion was body weight so that each selected dog fell into a defined weight range in order to ensure that dogs were dosed with whole tablet(s) to deliver between 0.75 and 1.11 mg/kg of MO, which is the approximate lower half of the tablet unit dose range. In study 1, 51 dogs and in study 2, 44 dogs were orally inoculated with approximately 300 infective *A. caninum* L3 once on day 0. All dogs that met the inclusion/exclusion criteria were weighed on day 6 and then randomized to one of the six (study 1) or four (study 2) treatment groups.

### Experimental hookworm inoculations

On day 0 of each study, dogs were inoculated with 300 infective L3 of *A. caninum* via oral gavage. The L3s were confirmed by morphology to be those of *A. caninum*. All dogs within each study were inoculated orally using the same inoculation method. Any calculations needed to determine inoculum volume were recorded. Dogs were checked for vomiting at 1 h ± 30 min post-inoculation. The *A. caninum* isolate used in each study (separate isolate for each study) was a field strain and had undergone zero passages. Each isolate used at each study site was sourced from a local naturally infected dog accessed by each USA study site.

### Randomization and treatment

All dogs were weighed using certified scales on the day before the scheduled treatment to confirm eligibility within protocol defined weight ranges. Eligible dogs were then randomized into the treatment groups in a completely randomized design.

All dogs were fed a small portion of a moist canned food just prior to their scheduled dosing time point. Treatments using the intended commercial solid oral dosing formulation (Credelio Plus; Elanco Animal Health) were administered in a time frame relative to inoculation so that effectiveness could be assessed primarily against immature larval (L4) or immature adult [L5] stages of *A. caninum*. Dogs were orally treated with whole tablets by pilling with the MO + lotilaner combination tablets on day 7 (to assess efficacy against L4 stages based on the life cycle) or on day 11 (to assess efficacy against immature adults [L5] based on the life cycle). All corresponding control groups received a vehicle control tablet on day 7 or day 11. In study 1, an additional treatment group was included using lotilaner as a mono product (Credelio™) and was administered to dogs on day 7 or day 11 to assess non-interference. Treatment day dog observations were performed just after dosing on each scheduled dosing day and at 1 and 2 h as well as 4 and 8 h to confirm that the oral solid dosing formulation had been accepted and that any abnormal health observations were documented.

### Necropsy/worm counts

All dogs treated on day 7 or 11 were euthanized and necropsied 5 days post-treatment in accordance with relevant laboratory procedures. Food was removed for an overnight fast the day prior to each scheduled necropsy date. The entire digestive tract was removed and processed in accordance with the relevant standard operating procedures of each study site and standard parasitological procedures. Since immature stages of *A. caninum* were being assessed, the intestinal contents were washed over a fine mesh sieve (#100; 0.15 mm aperture). Additionally, the small intestine was soaked for approximately 2 h in warm saline to facilitate the emergence of immature stages from mucosal tissues. Materials recovered from soaked intestines were similarly sieved for worm recovery. The worms recovered from each dog were preserved in 10% formalin prior to being counted and microscopically identified to species and stage.

### Variable classification

The primary variable evaluated was the total count of adult, immature L4 and immature adult stages of *A. caninum* recovered at each post-treatment necropsy day. Each treatment was administered in a time frame relative to inoculation so that effectiveness could be assessed primarily against immature L4 or immature adult stages of *A. caninum*. Efficacy against experimentally induced *A. caninum* populations was determined post-treatment for the MO + lotilaner combination or the lotilaner mono product by comparing the geometric mean total count with that in the corresponding vehicle control group. Based on the time interval between dosing and when dogs in each group were necropsied, for the L4 group, immature adults were expected to be present and adults would be present for the immature adult (L5) group based on the pre-patent period of *A. caninum*. Efficacy ≥ 90% and a statistically significant difference (*P* < 0.05, two-sided) between each vehicle control group and the corresponding MO + lotilaner treated groups was required to demonstrate effectiveness in the MO + lotilaner treated groups against the L4 or immature adult stages of *A. caninum*. As per VICH guidelines, a minimum of five specimens of any stage (total of immature L4, immature adult, mature adult) was required in a minimum of six control dogs at the end of each study to meet adequacy of infection criteria for each parasite stage of interest.

### Data analysis

For each dog the total *A. caninum* counts at necropsy were calculated as the sum of the immature L4, immature adult and adult counts. A logarithmic transformation (ln[count + 1]) was applied to the total post-treatment worm count for each individual animal to address the skewed nature of these data and also to allow zero counts. Back-transformed geometric means (GM) were calculated as (e^mean^ – 1) where the "mean" was the treatment group arithmetic mean of log-transformed counts at a given time point. The efficacy against each stage of *A. caninum* was calculated using the following formula: [(*C* − *T*)/*C*] × 100, where *C* was the geometric mean total worm count for the placebo group and *T* was the geometric mean total worm count for the treated group. The transformed counts were analyzed with a general linear model with fixed effect treatment and block as a random effect. In order to assess the effectiveness of the treatment against the immature L4 stage of *A. caninum*, statistical contrasts were constructed comparing the L4 control group against the L4 treated group in each of the studies. Similarly in each study, the immature adult control group and the immature adult treated group were compared to determine the effectiveness of the treatment against the immature adult stage [L5] of *A. caninum*. All statistical analyses used the statistical package SAS 9.4 (Cary, NC).

## Results

Based on daily observations to assess any adverse events, there were no mortalities and no treatment-related adverse reactions in either laboratory dose confirmation study. Effectiveness results against immature *A. caninum* stages are summarized in Table 1.

**Table 1:** Effectiveness of a single oral dose of a novel chewable tablet (Credelio Plus) containing milbemycin oxime and lotilaner against induced L4 larval and immature adult (L5) *Ancylostoma caninum* infections in dogs

Study	Stage at time of treatment	Day of inoculation ^a^	Day of treatment	Day of necropsy (worm recovery)	Treatment group	*n*	No. of infected dogs	Worm count range	Geometric worm count	Effectiveness compared to vehicle control	
										% efficacy	Effectiveness *P*-value
1	L4 Larvae	0	7	12	Vehicle control	8	8	60–248	171.9	–	–
					Credelio Plus^b^	8	8	1–15	4.6	97.3	*P* < 0.001
					Credelio^c^	8	8	98–236	171.7	0.1	*P* < 0.001
2	L4 Larvae	0	7	12	Vehicle control	10	10	42–156	79.4	–	–
					Credelio Plus^b^	10	3	0–2	0.3	99.6	*P* < 0.001
1	Immature adult (L5)	0	11	16	Vehicle control	8	8	173–255	198.7	–	–
					Credelio Plus^b^	8	6	0–86	2.5	98.7	*P* < 0.001
					Credelio^c^	8	8	149–231	178.8	10.0	*P* < 0.001
2	Immature adult (L5)	0	11	16	Vehicle control	10	10	61–302	109.7	–	–
					Credelio Plus^b^	10	2	0–3	0.3	99.7	*P* < 0.001

*n* Number of animals per group

^a^Each dog was inoculated with ca. 300 L3 *A. caninum*

^b^Credelio Plus provided minimum dosages of 0.845 to 0.943 mg/kg of milbemycin oxime in both studies

^c^Credelio™ (lotilaner mono product) was included in study 1 to assess non-interference

### L4 *A. caninum worm* counts: study 1

All eight dogs in the vehicle control group (treated on day 7) had positive *A. caninum* counts recorded at necropsy with a range of 60–248 worms (Table 1). This demonstrated a robust level of infection because all 8 of the control dogs were adequately infected with at least 60 worms. The geometric mean (GM) *A. caninum* count for the vehicle control group was 171.9. A small number of *A. caninum* were recovered from all 8 dogs in the Credelio Plus group (range 1–15). The GM *A. caninum* count for the Credelio Plus group was 4.6. The GM *A. caninum* count for the Credelio (Mono) group to assess non-interference of lotilaner was 171.7 (range 98–236). The effect of the Credelio Plus tablet on L4 *A. caninum* was clearly demonstrated at necropsy based on worm counts and as compared to the vehicle control group. Post-treatment *A. caninum* worm counts were significantly different (*P*-value < 0.001) between the Credelio Plus group compared to the vehicle control group. Non-interference between the active ingredients was also demonstrated because the counts in the Credelio Plus group were significantly lower than in the Credelio Mono group (*P* < 0.001) and efficacy in the Credelio Plus group exceeded 90% while efficacy in the Credelio Mono group was < 1%.

### Immature adult (L5) *A. caninum* worm counts: study 1

All eight dogs in the vehicle control group (treated on day 11) had positive *A. caninum* counts recorded at necropsy with a range of 173–255 (Table 1). This demonstrated a robust level of infection because all 8 of the control dogs were adequately infected with at least 173 worms. The GM *A. caninum* count for the vehicle control group was 198.7. *Ancylostoma caninum* were recovered from six of the eight dogs in the Credelio Plus group (range 0–86). The GM *A. caninum* count for the Credelio Plus group was 2.5. The GM *A. caninum* count for the Credelio Mono group was 178.8 (range 149–231).

The effect of the Credelio Plus tablet on immature adult (L5) *A. caninum* was clearly demonstrated at necropsy based on worm counts and as compared to the vehicle control group. *Ancylostoma caninum* worm counts were significantly reduced post-treatment from 198.7 in the vehicle control group to 2.5 in the Credelio Plus treated group (98.7% efficacy). Post-treatment *A. caninum* worm counts were significantly different (*P*-value < 0.001) between the Credelio Plus group compared to the vehicle control group. Non-interference between the active ingredients was also demonstrated because the immature adult (L5) counts in the Credelio Plus group were significantly lower than in the Credelio Mono group (*P* < 0.001) and efficacy in the Credelio Plus group exceeded 90% while efficacy in the Credelio Mono group was 10%.

### L4 *A. caninum* worm counts: study 2

All 10 dogs in the vehicle control group had positive *A. caninum* counts recorded at necropsy with a range of 42–156 (Table 1). This demonstrated a robust level of infection because all 10 of the control dogs were adequately infected with at least 42 worms. The GM *A. caninum* count for the vehicle control group was 79.4. In total, four *A. caninum* were recovered from three of the 10 dogs (two dogs had 1 worm, and one dog had 2 worms) in the Credelio Plus group. The GM *A. caninum* count for the Credelio Plus group was 0.3. The effect of the Credelio Plus tablet on L4 *A. caninum* was clearly demonstrated at necropsy based on worm counts and as compared to the vehicle control group. *Ancylostoma caninum* worm counts were reduced significantly post-treatment from 79.4 in the vehicle control group to 0.3 in the Credelio Plus treated group (99.6%). Post-treatment *A. caninum* worm counts were significantly different (*P*-value < 0.001) between the Credelio Plus group compared to the vehicle control group.

### Immature adult [L5] *A. caninum* worm counts: study 2

All 10 dogs in the vehicle control group had positive *A. caninum* counts recorded at necropsy with a range of 61–302 (Table 1). This demonstrated a robust level of infection because all 10 of the control dogs were adequately infected with at least 61 worms. The GM *A. caninum* count for the vehicle control group was 109.7. In total, six *A. caninum* were recovered from 2 of the 10 dogs (2 dogs with 3 worms each) in the Credelio Plus group. The GM *A. caninum* count for the Credelio Plus group was 0.3. The effect of the Credelio Plus tablet on the immature adult (L5) *A. caninum* was clearly demonstrated at necropsy based on worm counts and as compared to the vehicle control group. *Ancylostoma caninum* worm counts were reduced significantly from 109.7 in the vehicle control group to 0.3 in the Credelio Plus treated group (99.7%). Post-treatment *A. caninum* worm counts were significantly different (*P*-value < 0.001) between the Credelio Plus group compared to the vehicle control group.

## Discussion

The objective of both dose confirmation studies was to evaluate the effectiveness and safety of Credelio Plus (lotilaner + milbemycin oxime), administered once for the treatment of immature stages of the intestinal nematode species *A. caninum*. The two-dose confirmation studies summarised above clearly demonstrated that the Credelio Plus chewable tablets administered orally at approximately the lower end (0.75 to 1.11 mg/kg MO) of the proposed MO tablet unit dosage range (0.75 to 1.53 mg/kg MO) was highly efficacious when treating dogs that were infected with immature (L4 and immature adult [L5]) stages of *A. caninum* at the time of each treatment*.* In studies designed to treat immature *A. caninum*, worm counts were reduced by 97.3% to 99.7%, in both studies in comparison to a vehicle control group.

Larval stages of *A. caninum* are considered the dose-limiting intestinal nematode species for MO and a minimum MO dosage of 0.75 mg/kg was confirmed in these studies to demonstrate adequate and substantial evidence of effectiveness [10]. It was previously reported that slightly lower dosages of MO at 0.5 mg/kg did not demonstrate acceptable (> 90%) efficacy against larval (L4) or immature adult stages of this pathogenic hookworm species of dogs [29]. These authors reported that 0.5 mg/kg MO had 49% efficacy against L3/L4 stages, 83% against L4 and 81% against early (immature) adult stages of *A. caninum*. It is very important that administered doses of MO and other anthelmintics are accurate, based on the approved label dosage (mg/kg) and the dog’s body weight, because small variations could potentially impact the overall efficacy against different life cycle stages of pathogenic parasites such as *A. caninum*.

The ectoparasiticidal component of the IP, lotilaner, is already registered in the European Union (lotilaner, Credelio chewable tablets for dogs) for the treatment of flea (*Ctenocephalides felis* and *C. canis*) and tick (*Rhipicephalus sanguineus*, *Ixodes ricinus*, *Ixodes hexagonus*, and *Dermacentor reticulatus*) infestations in dogs, and additionally has been shown to be effective against other global tick and mite species [11–24]. To extend the effectiveness of the commercial drug product when dogs have concurrent nematode infections and ectoparasite infestations, lotilaner was combined with MO to form Credelio Plus. *Ancylostoma caninum* along with other intestinal nematode species is commonly reported from dogs globally as well as in Europe [2, 5, 6, 30, 31].

Since both the larval and adult stages of *A. caninum* are very pathogenic in dogs and are commonly diagnosed in the general dog population, this further warrants the need to routinely conduct periodic faecal examinations and to treat dogs for this intestinal nematode parasite. This includes dogs maintained in their home environment as well as pets that contact other dogs or visit places contaminated by dog feces because these circumstances may support transmission of parasites [32, 33]. The use of Credelio Plus as a broad spectrum endectocide that pet owners and veterinarians can use to effectively treat dogs with adult and immature intestinal nematode infections supports the recommendations from scientific expert groups such as ESCCAP and TroCCAP to provide regular treatment and control of all intestinal nematodes of dogs and cats [34, 35]. If *A. caninum* is a concern based on past infections or exposure to contaminated areas, deworming at least four times a year should be considered if dogs and cats are housed outside or have access to the outdoors. ESCCAP further points out that the routine treatment and prevention of all intestinal parasites depends upon legislation in individual countries, veterinary professionals taking local epidemiological circumstances into account, owner perception, and individual risk assessments (e.g., hunting pets, previous lungworm exposure, raw meat diets). Deworming practices should therefore always be on the advice of a veterinary professional. The use of Credelio Plus is of particular importance for zoonotic species like *T. canis* and the zoonotic and pathogenic hookworm species, *A. caninum.* Both are commonly reported in dogs of all ages and throughout the year in prevalence studies conducted in different areas of the world, including healthy, well-cared-for dogs in Europe [3, 4, 30–33, 36, 37]. There is surprisingly little information about the impact of re-treatment intervals on parasite burdens and environmental contamination on which to base a maximum re-treatment interval. Current information suggests that annual or twice-yearly treatments do not have a significant impact on preventing patent intestinal nematode infections within a population of dogs, so a treatment frequency of at least 3–4 times per year is a general recommendation that may reduce faecal egg shedding and environmental contamination [31]. Monthly deworming treatments can largely prevent patent infections as it takes into account the biology and pre-patent period of these different intestinal nematode parasites. It was shown that the prevalence of common nematode and cestode endoparasites declined significantly in a population of well-cared for dogs in which broad spectrum endectocides were used on a monthly basis [38]. The MO minimum dosage (0.75 mg/kg) in Credelio Plus additionally has been shown to effectively treat dogs with L4 and immature adult stages of both *T. canis* and *A. caninum* and for reducing the level of infection with the lungworm, *Angiostrongylus vasorum* and thereby also reducing environmental contamination [9, 10].

MO, used alone or in combination with other oral parasiticides, has been used safety for over 20 years for intestinal nematode control and heartworm prevention in dogs [39]. It has however been recently documented that there are multiple-drug resistant field isolates of adult *A. caninum* [40]. Lotilaner (Credelio) as a stand-alone product administered as an oral ectoparasiticide has demonstrated safety for dogs under both field use and laboratory conditions [11–24].

## Conclusions

These studies confirmed the efficacy of a single oral dosage of a novel, chewable tablet containing milbemycin oxime and lotilaner (Credelio Plus) against immature stages of *A. caninum* infections in dogs. This new combination treatment option of lotilaner + MO can be used to provide broad spectrum parasite control for dogs because it offers flea and tick prevention and control, intestinal parasite treatment and control, and heartworm and lungworm disease prevention. This will contribute to owner compliance and simplifies the treatment recommendations from global scientific groups and veterinarians to prevent or treat these parasites as well as decreasing the transmission of important zoonotic parasites and tick- and flea-transmitted disease agents.

## Data Availability

The dataset summarizing and supporting the conclusions of this article are included within the article. Due to commercial confidentiality of the research, data not included in the manuscript can only be made available to bona fide researchers subject to a fully executed non-disclosure agreement.

## Abbreviations

ESCCAP: European Scientific Counsel Companion Animal Parasites
GM: Geometric mean
IP: Investigational product
MO: Milbemycin oxime
TroCCAP: Tropical Counsel for Companion Animal Parasites
